# Statistical Manifold Generation-Driven Equipment Collaborative Personalized Fault Diagnosis

**DOI:** 10.3390/s26154696

**Published:** 2026-07-23

**Authors:** Kaiwei Liu, Yaowei Shi

**Affiliations:** 1 Portland Institute, Nanjing University of Posts and Telecommunications, Nanjing 210023, China; p23000611@njupt.edu.cn; 2 School of Internet of Things, Nanjing University of Posts and Telecommunications, Nanjing 210023, China

**Keywords:** fault diagnosis, unknown working condition, federated learning, domain generalization, statistical information

## Abstract

In Industrial Internet of Things (IIoT) scenarios, data privacy constraints and distribution discrepancies caused by time-varying working conditions hinder existing intelligent fault diagnosis models from maintaining robust generalization performance across different, particularly unknown, working conditions. To address this challenge, a federated generalization fault diagnosis method driven by statistical manifold generation is proposed. The method constructs a closed-loop collaborative strategy. Initially, individual users extract and upload representative statistical information (SI) as lightweight, privacy-preserving knowledge carriers. Subsequently, a global Gaussian mixture model coupled with a covariance expansion mechanism is established in the cloud to fit multi-source distributions and extrapolate uncertainty boundaries, thereby generating virtual SI. Finally, this virtual SI is assigned via a difference-aware mechanism and integrated locally using instance normalization to achieve domain-invariant augmented training. Extensive distributed collaborative fault diagnosis experiments conducted on rolling bearing and gearbox datasets demonstrate that, when facing completely unknown working conditions, the proposed method achieves an average diagnostic accuracy of over 85%, exceeding 90% in some tasks. Furthermore, the communication payload per round is merely 1.25 KB. While strictly preserving data privacy, the proposed method significantly enhances the cross-domain generalization capability of local models with minimal communication overhead, providing an efficient and robust collaborative intelligent diagnosis solution for resource-constrained IIoT edge devices.

## 1. Introduction

In recent years, with the rapid proliferation of the Industrial Internet of Things (IIoT) and significant improvements in equipment connectivity, data-driven intelligent fault diagnosis technology for mechanical equipment has made substantial progress. This has effectively enhanced the operational reliability and safety of industrial equipment while reducing maintenance costs [1,2]. However, current mainstream intelligent diagnosis methods are constrained by the assumption that training and test data are independent and identically distributed (i.i.d.), which leads to poor performance in cross working condition diagnosis tasks [3,4]. This limitation arises because data collected under different working conditions typically exhibit varying degrees of distributional differences. Particularly in time-varying scenarios, deployed models must diagnose equipment operating under unknown conditions that are not covered by the training data, which significantly increases the risk of misdiagnosis and missed detection [5,6].

To address the aforementioned issues, researchers have attempted to introduce domain adaptation and domain generalization techniques into the fault diagnosis field [7,8,9,10]. This technique aims to learn domain-invariant features related to faults, thereby enhancing the model’s adaptability to target domains [11]. Recent advances along this line have explored various directions, including prior knowledge-embedded first-layer interpretable paradigms for interpretable fault monitoring [12], integrated-dispersion manifold distance for distribution discrepancy measurement under time-varying conditions [13], and asynchronous joint distribution alignment for class-wise domain confusion [14]. However, despite achieving state-of-the-art performance, these methods fundamentally operate under domain adaptation or centralized paradigms. They either rely on centralized source data or require explicit access to the unlabeled target domain data during the training/adaptation phase. In practical industrial scenarios, data collection and annotation are costly, and a single user typically finds it difficult to complete this task independently [15]. Although it is theoretically feasible to collaboratively utilize local data from similar devices from multiple users for model training, data privacy requirements often prohibit raw data from leaving local storage [16]. Consequently, domain generalization methods based on centralized training exhibit significant limitations in practical applications [17].

Federated learning (FL) technology provides an effective approach for multi-user collaborative modeling [18,19,20,21]. By exchanging model weights or relevant knowledge, FL implicitly aggregates data from multiple users, thereby ensuring that each user’s local private data remain inaccessible to the cloud or other users. More challenging still, in practical diagnostic tasks, target devices often encounter entirely new working conditions (target domain) not encountered during training. Traditional federated transfer learning (FTL) or federated domain adaptation (FDA) typically assumes that target domain data (at least unlabeled data) are partially available during training. This assumption is clearly inapplicable in cold-start or real-time monitoring scenarios. Therefore, federated domain generalization (FDG) has been proposed, enabling models to learn robust and generalizable knowledge from distributed source domains under data privacy constraints without accessing target data. Currently, research on the FDG has primarily focused on fields such as computer vision and medical image analysis [22,23,24], while its application in fault diagnosis remains in the preliminary exploration stage [25,26].

Existing FDG explorations in fault diagnosis primarily unfold along four distinct directions: (1) domain alignment-based methods, which aim to mitigate inter-domain discrepancies by explicitly aligning data distributions or feature representations. For example, Fan et al. [27] proposed a federated fault diagnosis method based on prototype learning. By enforcing the alignment of prototype features across all users, this method enables the model to exhibit strong generalization capabilities when faced with changing working conditions. (2) Data manipulation-based strategies, which drive model generalization by synthesizing virtual samples to enrich data diversity. For example, reference [28] proposes an FL-based joint modeling framework. By performing weighted optimization on multi-user information and utilizing the principle of entropy minimization, this framework enhances the model’s adaptability under varying working conditions. (3) Learning strategy-based approaches, which focus on designing novel optimization paradigms to extract domain-invariant representations. For example, Wang et al. [29] propose a data-centric flatness optimization method to seek flat minima via global model-constrained adversarial data augmentation. (4) Aggregation optimization-based methods, which address data heterogeneity by robustly integrating local models to construct a generalizable global model. Within these frameworks, several representative studies have been conducted. For example, Qian et al. [30] propose a heterogeneous federated DG network, utilizing mutual information weight matching to optimize knowledge aggregation from heterogeneous sources.

A comprehensive review of existing FDG-based fault diagnosis research reveals that model parameter aggregation, feature space alignment, and data generation are currently the mainstream paradigms. However, a critical analysis of existing methods reveals specific bottlenecks when dealing with out-of-distribution (OOD) scenarios, data heterogeneity, and personalized modeling. Specifically, parameter aggregation-based methods inherently pursue a global consensus. Under highly heterogeneous industrial data distributions, this strategy hinders the model’s ability to retain local diagnostic characteristics, leading to suboptimal performance on client-specific data. Feature-alignment-based methods attempt to bridge the gap between domains, but their effectiveness relies heavily on the assumption that there is overlap between the feature spaces of the source and target domains. Under unknown working conditions, fixed alignment constraints cannot adapt to shifts in the feature manifold structure caused by changes in rotational speed or load. Even if the fault type itself remains unchanged, the distribution pattern of features for the same fault may change significantly under different working conditions, leading to alignment failure. In addition, data generation-based methods rely on synthesizing virtual samples from existing data distributions, which faces inherent challenges in that the generated samples are limited to the convex hull of the known source domain, making it difficult to effectively simulate statistical features of completely invisible working conditions, especially when the target domain is outside the distribution range covered by any participating client.

To address these challenges, this paper proposes a federated personalized fault diagnosis method driven by statistical manifold generation. The proposed framework addresses these limitations from three aspects. First, instead of transmitting model parameters or gradients, our method exchanges only lightweight statistical information (SI), reducing communication overhead while enhancing privacy. Second, rather than generating samples within known distributions, we construct a global Gaussian mixture model with covariance expansion to extrapolate the statistical manifold toward unseen working conditions. Third, a difference-aware assignment strategy and local instance normalization personalize the generated virtual SI to complement each client’s distributional gaps, mitigating the homogenization effect of global aggregation. These distinctions make the proposed method a solution that can simultaneously address OOD generalization, data heterogeneity, and personalized modeling within a privacy-preserving federated framework.

## 2. Collaborative Generalized Intelligent Diagnosis Problem Description

Consider a typical collaborative operations and maintenance scenario, in which identical types of machinery are deployed across multiple geographically dispersed industrial sites. These sites (i.e., users) face the following practical constraints:

(1) Operational heterogeneity (non-i.i.d. data): Due to variations in geographical conditions (temperature, humidity) and production tasks (load, rotational speed), monitoring data collected from different users, although containing the same fault modes, exhibit significant differences in distribution (i.e., domain shift).
(2) Data silos and privacy: To protect trade secrets or due to network bandwidth limitations, the transmission of raw, high-frequency monitoring data from individual sites to the cloud or other users is strictly prohibited; only the exchange of a limited amount of model parameters or statistical data is permitted.
(3) Requirements for generalization under unknown working conditions: In actual operation, equipment may encounter entirely new working conditions that none of the participating users have ever experienced (such as extreme weather or overload conditions). A diagnostic model must not only perform well under known working conditions but also possess the ability to generalize and make inferences about completely unknown conditions.

Based on the aforementioned scenario, this paper defines the collaborative generalized intelligent diagnosis problem as follows: Suppose there are *K* users (source domains), where *D_k_* denotes the private dataset of the *k*-th user, comprising *N_k_* data points. (*x_k_*_,*i*_, *y_k_*_,*i*_) represents the *i*-th data sample and its label in *D_k_*. Let *Z* denote the number of health states contained in the data. Due to differences in working conditions, the data distributions of any two users are distinct from each other. The objective of this paper is to design a collaborative learning strategy that utilizes data from *K* source domains for joint training, thereby improving the generalization performance of each local model while satisfying data privacy constraints. Each local model must not only perform well on its local test set but also, more importantly, maintain a high fault diagnosis accuracy when faced with a completely new target data distribution that was never encountered during the training phase.

## 3. Federated Generalization Fault Diagnosis Method

The federated domain generalization fault diagnosis method (FedGen) proposed in this paper primarily consists of three components: supervised learning of the local model, statistical manifold generation and personalized SI assignment, and local model enhancement training.

**Figure 1:**
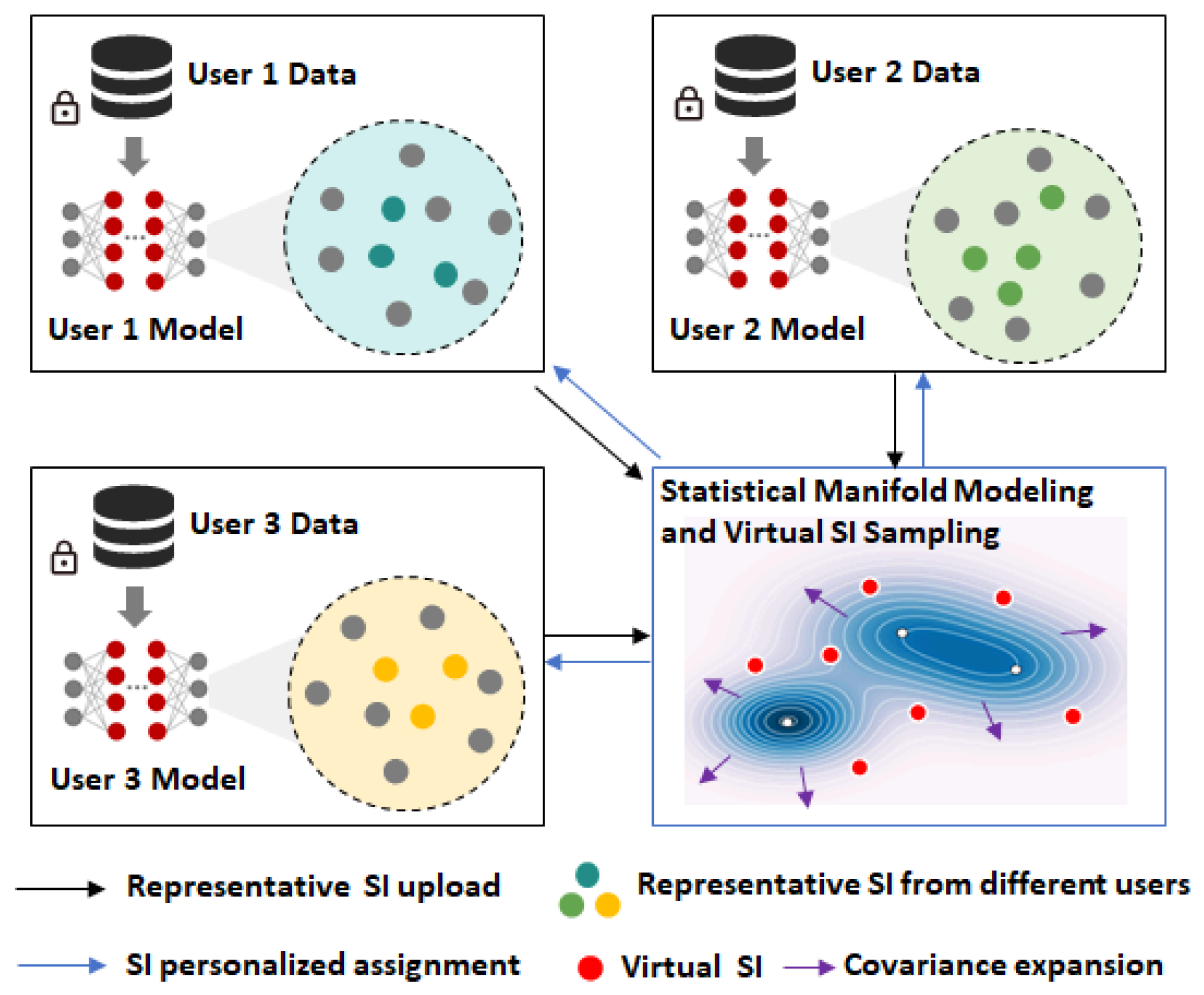
Schematic diagram of the proposed FedGen.

The execution pipeline of FedGen proceeds as follows: First, each user extracts local SI to parameterize the current data distributions. The cloud then constructs a global statistical manifold using all uploaded SI and performs generative sampling with covariance expansion to create virtual SI that simulates unknown working conditions. Next, a difference-aware assignment strategy distributes this virtual SI to users according to their local distribution gaps. Finally, users apply instance normalization to fuse the received SI with original features, executing enhancement training to build robust decision boundaries against unknown working conditions. The proposed FedGen is illustrated in Figure 1.

### 3.1. Supervised Learning of the Local Model

In the local model training for each user, supervised learning is performed using cross-entropy loss l(⋅,⋅) based on private annotated data, which provides a standard and effective classification objective for the local feature encoder. For example, the optimization objective for training the *k*-th user model is defined as:

(1) $$\begin{array}{l} \min L_{k}=-\sum_{x_{k,i}\in D_{k}}l\left(P_{k}\left(E_{k}\left(x_{k,i}\right),y_{k,i}\right)\right)\\ \;\;\;\;-\sum_{i=1}^{N_{k}}\sum_{z=1}^{Z}1\left\{y_{k,i}=z\right\}\ln\frac{\exp\left(x_{k,i,z}\right)}{\sum_{j=1}^{Z}\exp\left(x_{k,i,j}\right)} \end{array}$$

where *N_k_*, *x_k_*_,*i*_, and *y_k_*_,*i*_ denote the number of samples, the *i*-th data sample, and its category label, respectively, and E*_k_* (·) and P*_k_* (·) represent the feature encoder and fault category predictor of the *k*-th user model, respectively.

Through the supervised learning process, the user’s local model gains an initial ability to distinguish local data, providing the feature foundation for subsequent SI extraction and generative augmentation.

### 3.2. Statistical Manifold Generation and SI Personalized Assignment (SMGPA)

According to the theory of Instance Normalization (IN) [31], the distribution statistics (mean and standard deviation) of feature maps in deep neural networks primarily encode domain-specific information (i.e., distribution shifts induced by varying working conditions and acquisition environments). Meanwhile, the normalized feature maps preserve the condition-invariant essential structures for fault discrimination. Motivated by this, the core idea of FedGen is to jointly model and generatively extrapolate SI in the feature space, then inject the generated virtual SI into local features via IN. This achieves feature-level augmentation for potential unknown conditions without sharing raw data or model weights, preserving privacy while enabling personalization.

Specifically, the proposed SMGPA strategy consists of four steps: local SI extraction, representative SI mining, virtual SI generation, and SI personalized assignment.

#### 3.2.1. Local SI Extraction

In the field of fault diagnosis, there is already a relevant research foundation for encoding domain/style information using feature statistics (such as mean and standard deviation) [32,33]. From a physical perspective, changes in equipment working conditions (speed, load) can alter the amplitude level and spectral structure of vibration signals. These changes, when projected into the deep feature space, are correspondingly reflected in the concentration and dispersion of feature distributions. Therefore, using feature statistics as carriers of domain information (speed, load) is feasible in the field of fault diagnosis. Furthermore, although higher-order statistics can provide a more detailed description of data distributions, they are sensitive to noise and computationally intensive, which would significantly increase the burden in resource-constrained IIoT scenarios. Therefore, this work selects the mean and standard deviation as lightweight domain information carriers while ensuring diagnostic performance, thereby achieving a reasonable balance between characterization capability and system efficiency. Specifically, the mean and standard deviation of each batch of features output by the local model’s feature encoder are first calculated to obtain the SI of the local data:

(2) $$\mu_{k,z}=\frac{1}{B_{k,z}}\sum_{b=1}^{B_{k,z}}F_{k,z}$$

(3) $$\sigma_{k,z}=\sqrt{\frac{1}{B_{k,z}}\sum_{b=1}^{B_{k,z}}{\left(F_{k,z}-\mu_{k,z}\right)}^{2}}$$

where **F***_k_*_,*z*_ denotes the model’s output features for category *z* samples within the *k*-th user’s training batch (containing *B_k_*_,*z*_ samples), and *μ_k_*_,*z*_ and *σ_k_*_,*z*_ represent the mean and standard deviation, respectively.

#### 3.2.2. Representative SI Mining

Given that uploading all SI data obtained during local training to the cloud would result in significant data redundancy and increase communication load, each user first stores the SI data extracted from their private data locally, forming a set of candidate SI, *Q*. Then, SI exemplars (SIe) that effectively represent the local data distribution are selected from *Q* to form a set *P* (*P* ⊆ *Q*), and *P* is uploaded to the cloud.

Specifically, to ensure the validity of SIe, an objective function is constructed to evaluate the subset quality:

(4) $$\underset{P\subseteq Q,\;\left|P\right|\leq m_{up}}{\max}S_{up}=\frac{\sum_{q_{i}\in Q,p_{j}\in P}k\left(q_{i},p_{j}\right)}{\left|Q||P\right|}-\frac{\sum_{p_{i},p_{j}}k\left(p_{i},p_{j}\right)}{{\left|P\right|}^{2}}$$

where k(a,b)=exp(−γ||a−b||) denotes the Radial Basis Function (RBF) kernel, and *m*_up_ represents the total number of SIe within the uploaded set *P*. While finding an exact solution to the combinatorial maximization in Equation (4) is computationally NP-hard, it has been proven [34] that the greedy algorithm provides a near-optimal solution with a theoretical lower bound for any normalized monotone submodular function. Given that the RBF-induced objective function inherently possesses this monotone submodular property [35], a greedy strategy is utilized for maximization, which iteratively identifies the optimal sample from the candidate set *Q*. Compared with random sampling or clustering-based selection, greedy optimization directly maximizes the submodular objective with guaranteed approximation quality.

#### 3.2.3. Virtual SI Generation

In real-world industrial settings, the operating parameters of mechanical equipment (such as rotational speed and load) typically exhibit continuous variation, which leads to a continuous shift in the distribution of monitoring data. To address the limitation that limited monitoring data cannot fully characterize the working condition space, FedGen uses the SIe sequences uploaded by each user to fit a statistical manifold of multi-source working conditions via a Gaussian Mixture Model (GMM), and introduces a covariance expansion mechanism to reasonably extend the uncertainty boundaries of the distribution. Based on this, the process of sampling virtual SI from the GMM essentially constitutes generative interpolation and extrapolation along the known feature manifold structure. This mechanism actively simulates changes in statistical characteristics driven by the evolution of working conditions. The generated virtual SI not only bridges the gaps between discrete working conditions but also effectively covers potential distribution blind spots, thereby enabling in-depth exploration of unknown working conditions. It should be noted that we adopted the GMM to model the distribution of the uploaded statistical information, primarily based on the following considerations. First, the GMM already has a broad application base in the field of fault diagnosis and offers good interpretability. Second, compared to generative models (VAE/GAN), the GMM, as a parametric density estimation method, requires only estimation of means and covariances without additional training, matching the computational constraints of IIoT edge devices.

To simplify the notation, the subscript *z* denoting category in the formula has been temporarily omitted here. It is assumed that multiple users upload a total of *N_up_* SIe. Since the standard deviation must be positive, a logarithmic transformation is first applied to it.

(5) $$\eta_{k}=\log\left(\sigma_{k}\right)$$

Second, *G* (≥*K*) Gaussian components are used for modeling:

(6) $$f\left(\mu ,\eta \right)=\sum_{g=1}^{G}\pi_{g}N\left(\left[\begin{array}{c} \mu \\ \eta \end{array}\right]\left|\left[\begin{array}{c} m_{\mu ,g}\\ m_{\eta ,g} \end{array}\right],\Sigma_{g}\right.\right)$$

where *π_g_* denotes the mixture weight of the *g*-th component, satisfying $\sum_{g=1}^{G}\pi_{g}=1$, and ${\left[m_{\mu ,g}\;\;m_{\eta ,g}\right]}^{⊤}$ and $\Sigma_{g}$ represent the mean vector and covariance matrix of the *g*-th component, respectively. Here, we adopt a diagonal covariance matrix for each Gaussian component to guarantee numerical stability and computational efficiency in the high-dimensional SI space. Furthermore, the GMM is optimized via the Expectation-Maximization algorithm (with log-likelihood convergence tolerance (10^−3^), a maximum of 100 iterations, and a regularization term (10^−6^) added to the diagonal elements to prevent numerical singularity).

To break through the limitations of existing data distributions and simulate more extreme working condition fluctuations, a covariance expansion mechanism is developed in FedGen. By setting the expansion coefficient *λ* (*λ* > 1), a scale rescaling is applied to the estimated Gaussian component covariance matrix, i.e., ${\Sigma^{\prime }}_{g}=\lambda \cdot \Sigma_{g}$. Physically, fluctuations in rotational speed or load induce changes in vibration responses, which further manifest as shifts in data distributions and an increase in feature uncertainty [36]. Therefore, introducing a scalar *λ* > 1 to amplify the covariance is equivalent to extrapolating outward along the manifold structure, aiming to simulate the feature uncertainty caused by operational fluctuations. By expanding the effective coverage range of the feature distribution, this mechanism covers potential distribution shift directions with a higher probability. Importantly, this extrapolation is anchored in real data: the covariance matrix $\Sigma_{g}$ is estimated from the actual source-domain SI uploaded by all participating users, ensuring that the expansion originates from physically meaningful distributions. Moreover, scaling the covariance matrix strictly preserves its principal eigenvectors, meaning the generated virtual SI retains the inherent correlation structure of the original data while proportionally expanding along these physically meaningful directions. Consequently, this mechanism is not an arbitrary mathematical manipulation of the latent space, but a controlled expansion that preserves the intrinsic structure of the real data, safely extending toward regions where plausible unseen working conditions may exist. Based on this, a category distribution *g* ~ Categorical (*π*_1_, *π*_2_, …, *π_g_*) is constructed using the prior mixing weights of each component, and the target component is selected through random sampling to generate the virtual SI representing the unknown working condition.

(7) $$\left[\begin{array}{c} \mu_{\mathrm{new}}\\ \eta_{\mathrm{new}} \end{array}\right]\sim N\left(\left[\begin{array}{c} m_{\mu ,g}\\ m_{\eta ,g} \end{array}\right],\Sigma_{g}\right)$$

Then, the standard deviation in logarithmic form is converted back to $\sigma_{\mathrm{new}}=\exp(\eta_{\mathrm{new}})$, yielding the virtual SI set U.

Based on the error upper bound theory for domain adaptation [37], the above strategy actively expands the support set of the source domain. By extending the manifold boundary to cover potential unknown domains, it effectively reduces the error upper bound between the source and target domains, thereby ensuring improved generalization performance.

#### 3.2.4. Personalized SI Assignment

Furthermore, FedGen dynamically selects and assigns novel statistical information (SI) that complements each user’s existing data distribution. This difference-aware assignment strategy not only effectively reduces redundant communication overhead, but also accurately fills the distribution gaps of individual users in the local feature space, thereby achieving a synergistic improvement in both personalized fitting of the local model and global generalization ability.

The specific procedure is as follows: select a virtual SI set *U_s_* (*U_s_* ⊆ *U*) from *U* for distribution. Let *Q_U_* represent the union of the user’s own SI and the SI distributed in previous training rounds. Then, use a greedy algorithm to select *U*_s_ that maximizes Equation (8):

(8) $$\begin{array}{l} \underset{U_{s}\subseteq U,\left|U_{s}\right|\leq m_{new}}{\max}S_{new}=\sum_{u_{s,i}\in U_{s}}\left[\frac{\sum_{u_{i}\in U}k\left(u_{s,i},u_{i}\right)}{\left|U\right|}-\frac{\sum_{q_{u,i}\in Q_{U}}k\left(u_{s,i},q_{u,i}\right)}{\left|Q_{U}\right|}\right]\\ \;\;\;\;\;\;+\log\;\det U_{s} \end{array}$$

The objective consists of three terms with distinct functions. First, the left-hand bracket promotes representativeness, ensuring that the selected virtual SI adequately captures the diversity of the global virtual SI pool. Second, the right-hand bracket enforces distinctiveness from the user’s existing SI, avoiding redundancy and encouraging complementarity. Third, the log-determinant regularization term encourages diversity among the selected SI itself. This criterion naturally promotes diversity within the selected subset, as the log-determinant increases when the selected samples are more dissimilar. Compared with entropy-based measures that primarily characterize the spread of a single distribution, the log-determinant regularizer is more directly aligned with the objective of selecting a mutually complementary set of virtual SI.

### 3.3. Local Model Enhancement Training

During the local model enhancement training, the received virtual SI is first fused with the original local features using the Instance Normalization (IN) mechanism to generate augmented features ${F^{\prime }}_{k,z}$ that incorporate the attributes of new working conditions, thereby effectively enriching the distributional diversity of the local feature space.

(9) $${F^{\prime }}_{k,z}=\mu_{\mathrm{new}}\cdot \frac{F_{k,z}-\mu_{k,z}}{\sigma_{k,z}}+\sigma_{\mathrm{new}}$$

Then, supervised enhancement training is conducted using the augmented features described above (as in Equation (1)), encouraging the model to extract fault essence representations that are independent of specific working conditions, thereby enhancing its generalization ability when faced with data distribution shifts. In addition, while covariance expansion effectively simulates unseen domains, variance extrapolation may inadvertently push the generated virtual features across class decision boundaries, causing the model to learn ambiguous or incorrect fault characteristics. To avoid semantic drift caused by feature augmentation and ensure high discriminability of the model, a consistency constraint is further imposed between the prediction outputs of the original features and the augmented features. This constraint aims to force the model to maintain a stable decision boundary under working condition disturbances, ensuring the predictive consistency of diagnostic results. It is defined as follows:

(10) $$\min L_{\mathrm{JS}}=\frac{1}{N}\sum_{i=1}^{N}\mathrm{JS}\left(\rho_{i}\left\|\bar{\rho }\right.\right)\;\;,\;\;\bar{\rho }=\frac{1}{N}\sum_{i=1}^{N}\rho_{i}$$

where *ρ_i_* denotes the predicted probability vector output by the model, $N=B_{k}\cdot \left(\right|U_{s}\left|+1\right)$ represents the total number of original and augmented samples in each training batch, and JS (·) denotes the Jensen–Shannon divergence.

### 3.4. FedGen Method Execution Process

**Figure 2:**
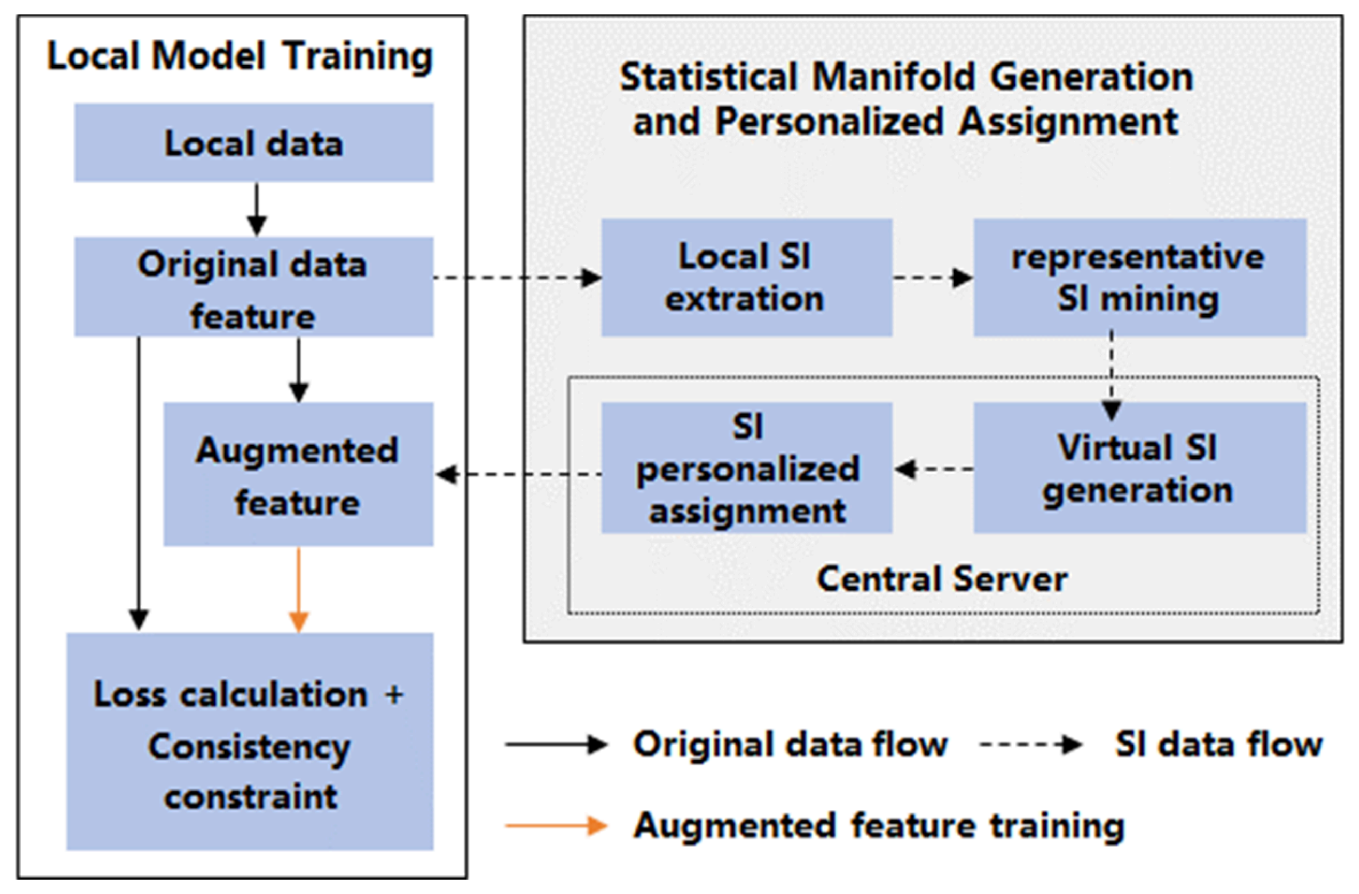
Process of local model training and cloud interaction.

Taking a specific local model as an example, the local model training and cloud interaction process in FedGen are illustrated in Figure 2.

Given that the feature extractor has not yet been fully optimized during the early stages of model training, the statistical information (SI) obtained lacks sufficient representativeness and stability. Moreover, overly frequent edge-to-cloud interactions would result in significant communication overhead.

To balance feature quality and communication costs, a periodic interaction strategy is adopted. Each user first trains independently for five epochs using local data, and then extracts and uploads the SI after the feature space has initially stabilized. Thereafter, a complete interaction process is triggered and executed every 2 training epochs.

The complete training procedure is summarized in Algorithm 1.
**Algorithm 1** Train details of FedGen**Input:** *K* clients, local datasets, total epochs *E*_max_, communication interval *E*, warm-up epochs *E*_warm_, total communication rounds *R*, expansion coefficient *λ*, upload count *m*_up_, assign count *m*_new_.**Output:** Trained local models.1:  Initialize global GMM; initialize local encoders E*_k_* (·) and classifiers P*_k_* (·).2:  **for** epoch *e* = 1 to *E*_max_ **do**:3:          **for** each client *k* in parallel **do**:4:                  Train (E*_k_*, P*_k_*) on *D_k_* via Equation (1).5:                  Extract and cache SI via Equations (2) and (3).6:          **end for**7:          **if** (*e* > *E*_warm_) and (*e* % *E* == 0) and (comm_round < *R*) then:8:                // Cloud-side: GMM fitting and virtual SI generation9:                Each client uploads representative SI subset via Equation (4).10:                Server fits GMM on aggregated SI via Equations (5) and (6).11:                Server expands covariance by *λ* and samples virtual SI set *U* via Equation (7).12:                // Client-side: personalized assignment and enhancement13:                Each client receives complementary virtual SI subset via Equation (8).14:                Each client augments local features via IN (Equation (9)).15:                    Each client trains with original + augmented features using JS loss (Equation (10)).16:                comm_round ← comm_round + 1.17:          **end if**18:  **end for**19:  return trained local models.

## 4. Dataset Introduction and Experimental Setup

### 4.1. Dataset Introduction

Two sets of experimental tasks are constructed using rolling bearing and gearbox datasets to validate the effectiveness of the proposed method.

**Figure 3:**
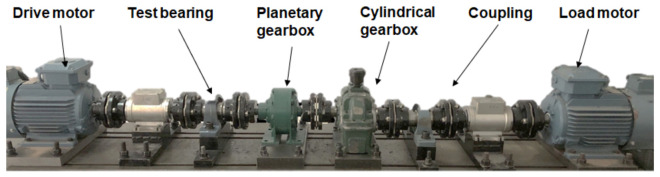
Bearing failure simulation test bench.

Dataset A: Figure 3 shows the rolling bearing failure simulation test bench. The experimental bearing is a UCP210 deep groove ball bearing with an inner diameter of 25 mm, an outer diameter of 90 mm, and a ball diameter of 12.7 mm. The collected data include five health states: normal condition, inner ring damage, outer ring damage, rolling element damage, and mixed inner/outer ring damage faults. The inner/outer ring damage refers to cracks introduced by electrical discharge machining, with crack widths ranging from 0.5 to 0.8 mm. State labels are denoted as 0–4. Vibration data was collected under four distinct operating speeds (500, 700, 800, and 1000 r/min), denoted as A1, A2, A3, and A4, respectively.

Dataset B: The experimental data were obtained from a transmission fault simulation test bench, which primarily consists of a drive AC motor, an input shaft, a test transmission, and a brake. The test transmission includes a pair of driving and driven gears. There are six health states in total: normal, driven gear pitting and tooth breakage, pinion surface wear, a composite failure involving both driven gear pitting and pinion surface wear, and a composite failure involving both driven gear tooth breakage and pinion surface wear. These states are labeled 0–5. Vibration data were collected under four working conditions, involving rotational frequency (Hz) and load (W), with a sampling frequency of 5120 Hz. The data from the four different working conditions are denoted as B1, B2, B3, and B4, respectively.

**Figure 4:**
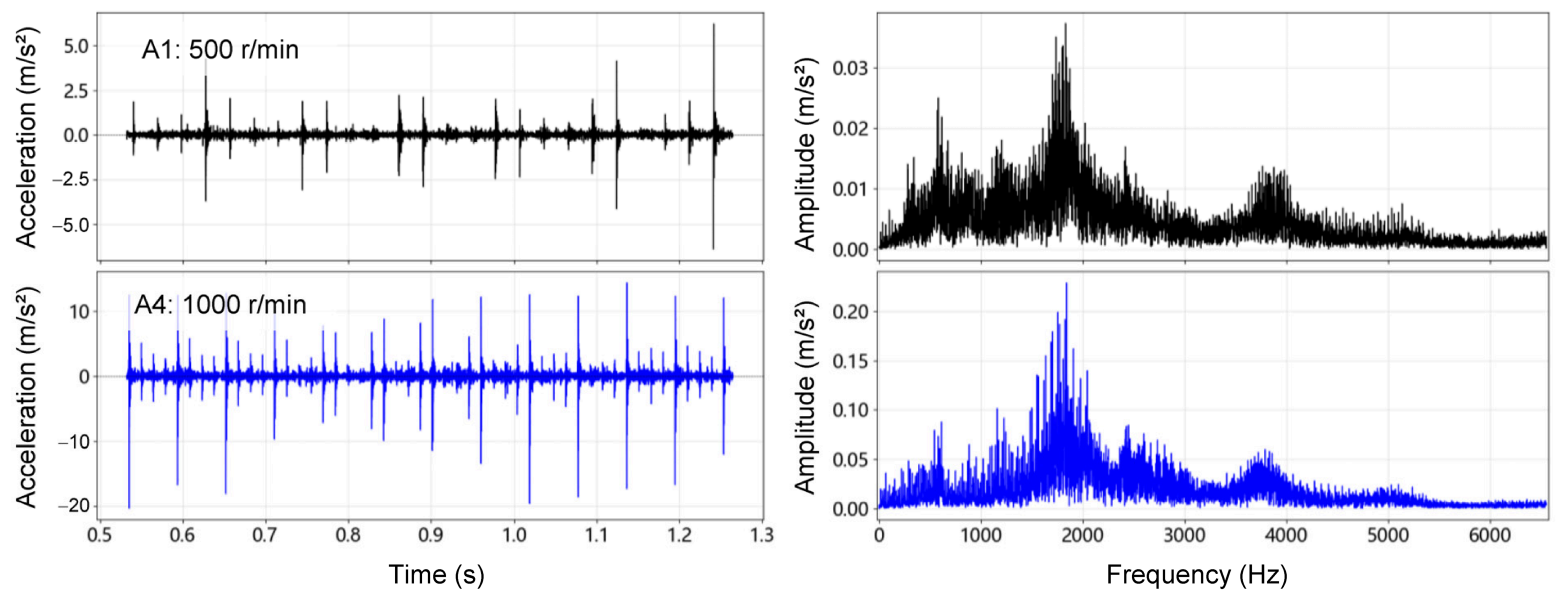
Time-domain and spectral comparison of outer ring damage under different rotational speeds.

Taking the outer ring damage in Dataset A as an example, Figure 4 presents the time-domain waveforms and FFT spectra of vibration signals under two rotational speeds: A1 (500 r/min) and A4 (1000 r/min). The time-domain waveforms show that the impulse trains in A4 are significantly denser and exhibit a much larger amplitude range compared to A1. This is attributed to the proportional relationship between fault characteristic frequency and rotational speed, as well as the substantial increase in impact energy at higher speeds. In terms of spectrum, the spectral amplitude of A4 is significantly higher than that of A1, consistent with the difference in time-domain impulse energy. These results confirm that speed variations induce systematic changes in both temporal and spectral characteristics of vibration signals, which constitute the cause of cross-condition domain shifts.

### 4.2. Fault Diagnosis Tasks and Related Experimental Setup

Based on the two datasets described above, the multi-user collaborative fault diagnosis tasks are constructed. Details are shown in Table 1 and Table 2.

The raw vibration signals for each health state were segmented using an overlapping sliding window strategy: the window length was fixed at 2400 data points, with an overlap of 200 data points between adjacent windows. A total of 500 independent samples were generated for each category via this strategy. Subsequently, samples of each category were randomly split into training and test sets at a 7:3 ratio. It should be noted that the training and test sets were generated from separate, independent windowing processes, with no sample overlap between them, thus avoiding data leakage. Based on the scenario settings for the collaborative diagnosis task, data collected under different working conditions is assigned to different users within the same task. Taking Task T1 as an example, the local training data for user 1, user 2, and user 3 is taken from working conditions A1, A2, and A3, respectively. Each user’s testing task consists of the following two parts.

(1) Cross-user testing task (denoted as T-cro, validating knowledge sharing effectiveness): The test set in this task comprises a mix of test data from all users except the local user. It aims to evaluate how effectively diagnostic knowledge is transferred among multiple users. For example, for the user 1 model, evaluation is conducted using a mix of test data from working conditions A2 and A3 (sample size: 300 samples per category). It is denoted as (A2, A3) in diagnostic task tables.
(2) Unknown working condition testing task (denoted as T-un, verifying generalization robustness): To simulate more challenging diagnostic scenarios, this paper introduces additional unknown working condition data that none of the users encountered during the training phase, in order to verify the generalization capabilities of each user model in handling domain shifts. For example, using the data from A4 (with 150 samples per category), test the models for user 1, user 2, and user 3.

Because FedGen uses SI as the carrier for knowledge exchange rather than directly sharing or aggregating model parameters, it allows different users to build deep neural networks tailored to their local data characteristics and hardware configurations. However, to eliminate the interference caused by additional variables introduced due to differences in model structure, and thereby objectively and fairly verify the effectiveness of the proposed method, all users adopted the same model structure. The specific parameters are as follows: Conv(32, 7)-MaxP(2)-Conv(64, 5)-MaxP(2)-Conv(64, 3)-MaxP(2)-Lin(512)-Lin(100)-Lin(*Z*). Here, Conv(32, 7) indicates a convolutional layer containing 32 convolutional kernels of size 7, MaxP(2) denotes a max-pooling layer with a stride of 2, Lin(128) represents a linear layer with 128 neurons, and so on.

The model training parameters are set as follows. For each user’s local model training, the stochastic gradient descent optimizer is used with a momentum of 0.9 and a learning rate decay of 5 × 10^−4^. The initial learning rate *η* is set to 0.01, and the batch size is set to 64. After 5 training epochs, the local model begins calculating SI data and uploading them to the cloud. It then communicates with the server once every 2 training epochs (i.e., the communication interval *E* is 2), with a total of 50 communication rounds. In addition, the number of sets of uploaded representative SI is *m*_up_ = 5, the number of GMM components is *G* = 3, and the number of sets of assigned virtual SI is *m*_new_ = 5 (for a fixed number of amplifications, in a total of 3 batches). All of these values were obtained through the sensitivity analysis experiments described in Section 5.4. Each experiment is repeated ten times, and the average value is recorded. All experiments are conducted with a fixed random seed (seed = 42) to ensure reproducibility.

### 4.3. Comparative Method

To fully validate the effectiveness and superiority of the proposed method, the following representative intelligent fault diagnosis methods were selected for comparison:

Local: Each user trains local models using only private data, without exchanging knowledge or collaborating with other users.

Central: A traditional centralized training method. All users’ data are aggregated for model training, and this approach cannot prevent data privacy breaches. Since all users’ data are used during training, the model is evaluated during testing using only data from unknown working conditions.

FedAvg: One of the current mainstream privacy-preserving distributed learning paradigms [38]. Each user trains a model independently using local data, and a cloud server periodically collects the model parameters (or increments) from each user and performs an averaging operation to update the global model.

LFDGN [39]: LFDGN is an FDG fault diagnosis method for addressing data heterogeneity. It employs a dual-level prototype-based interaction strategy to learn client-invariant representations for domain generalization.

FDDG [27]: FDDG is a federated distillation-based FDG method that leverages knowledge distillation to capture domain-invariant representations across clients.

To ensure the fairness and objectivity of the comparative experiments, all the above comparison methods adopted the same network architecture as FedGen and maintained consistent hyperparameter optimization settings overall.

## 5. Analysis of Experimental Results

### 5.1. Test Accuracy Analysis

**Figure 5:**
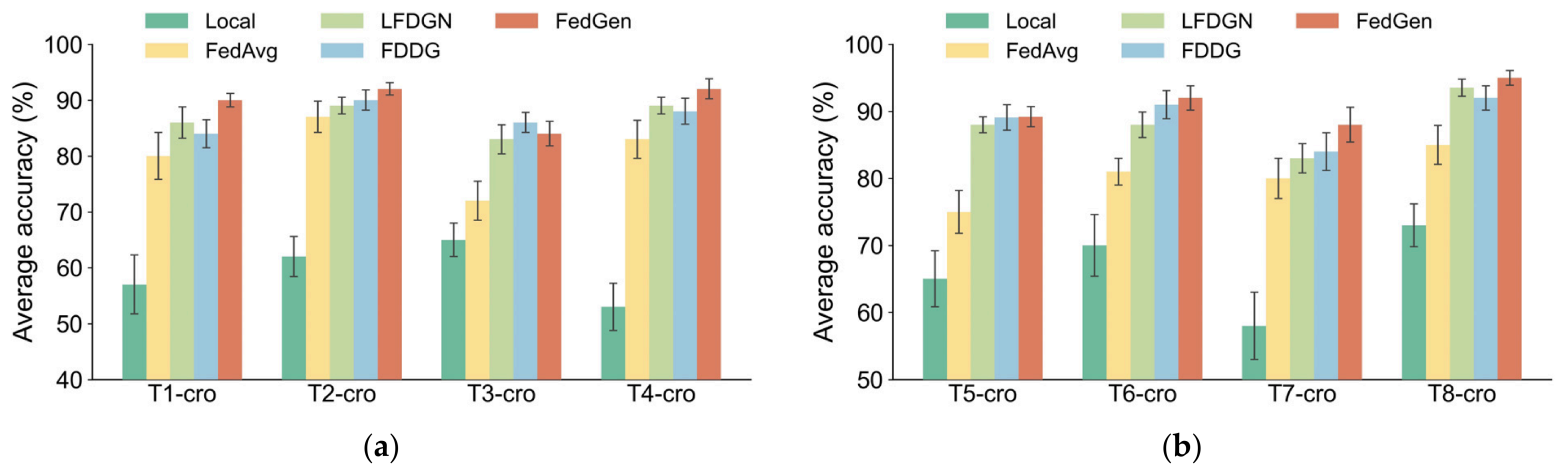
Multi-user average test accuracy across different methods in diagnostic tasks (cross-user testing task): (**a**) diagnostic task based on dataset A; (**b**) diagnostic task based on dataset B.

**Figure 6:**
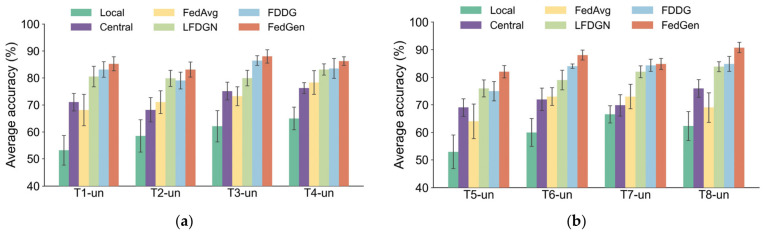
Multi-user average test accuracy across different methods in diagnostic tasks (unknown working condition testing task): (**a**) diagnostic task based on dataset A; (**b**) diagnostic task based on dataset B.

Figure 5 and Figure 6 show the test accuracy of FedGen and the various comparison methods on diagnostic tasks across the two datasets. To intuitively assess the overall collaborative diagnostic performance, the multi-user average accuracy (i.e., the mean of the test accuracies of all participating users for the same task) was adopted as the evaluation metric. Specifically, Figure 5 and Figure 6 correspond to (1) the cross-user testing scenario and (2) the unknown working condition testing scenario described in Section 4.2, respectively.

The experimental results show that the proposed FedGen method achieved the best generalization performance across all diagnostic tasks, with test accuracy consistently exceeding 80% (and surpassing 90% for some tasks), fully validating the method’s effectiveness and superiority in addressing distribution shifts.

In contrast, all comparison methods exhibit varying degrees of limitations. The Local method performs poorly across all tasks because it relies solely on each user’s local data to train the model. As a result, the model is highly vulnerable to local overfitting and struggles to adapt to distribution discrepancies between the test data and the training data, leading to low accuracy. By leveraging the advantages of multi-source data, the Central method achieves significantly higher accuracy than the Local method under unknown conditions (as shown in Figure 6). However, due to the limitations of the traditional empirical risk minimization strategy, it remains difficult to achieve ideal generalization performance when faced with completely unseen out-of-distribution (OOD) data. Although the FedAvg method implicitly leverages multi-user data through parameter aggregation and outperforms the Local method, its ability to handle working condition fluctuations remains limited due to the lack of explicit alignment or generalization mechanisms for distribution heterogeneity. In contrast, LFDGN employs a dual-level prototype contrastive learning strategy to explicitly align feature distributions across users. As a result, it performs exceptionally well on cross-user testing tasks (as shown in Figure 5). However, its accuracy drops significantly in testing tasks under unknown working conditions (as shown in Figure 6), because the prototype alignment mechanism is strictly confined to the existing domain and struggles to generalize to out-of-distribution scenarios. FDDG, which adopts a federated distillation strategy, achieves competitive performance on cross-user tasks and several unknown working condition tasks. Nevertheless, its overall accuracy remains below that of FedGen across most tasks. It further validates the effectiveness of the proposed statistical manifold generation and boundary extrapolation strategy for exploring unknown working conditions and achieving reliable generalization.

Benefiting from enhanced training of virtual features, FedGen introduces adaptive learning to address potential distribution shifts while strictly preserving the integrity of existing data discriminative information, thereby significantly improving the model’s robustness and generalization performance.

**Figure 7:**
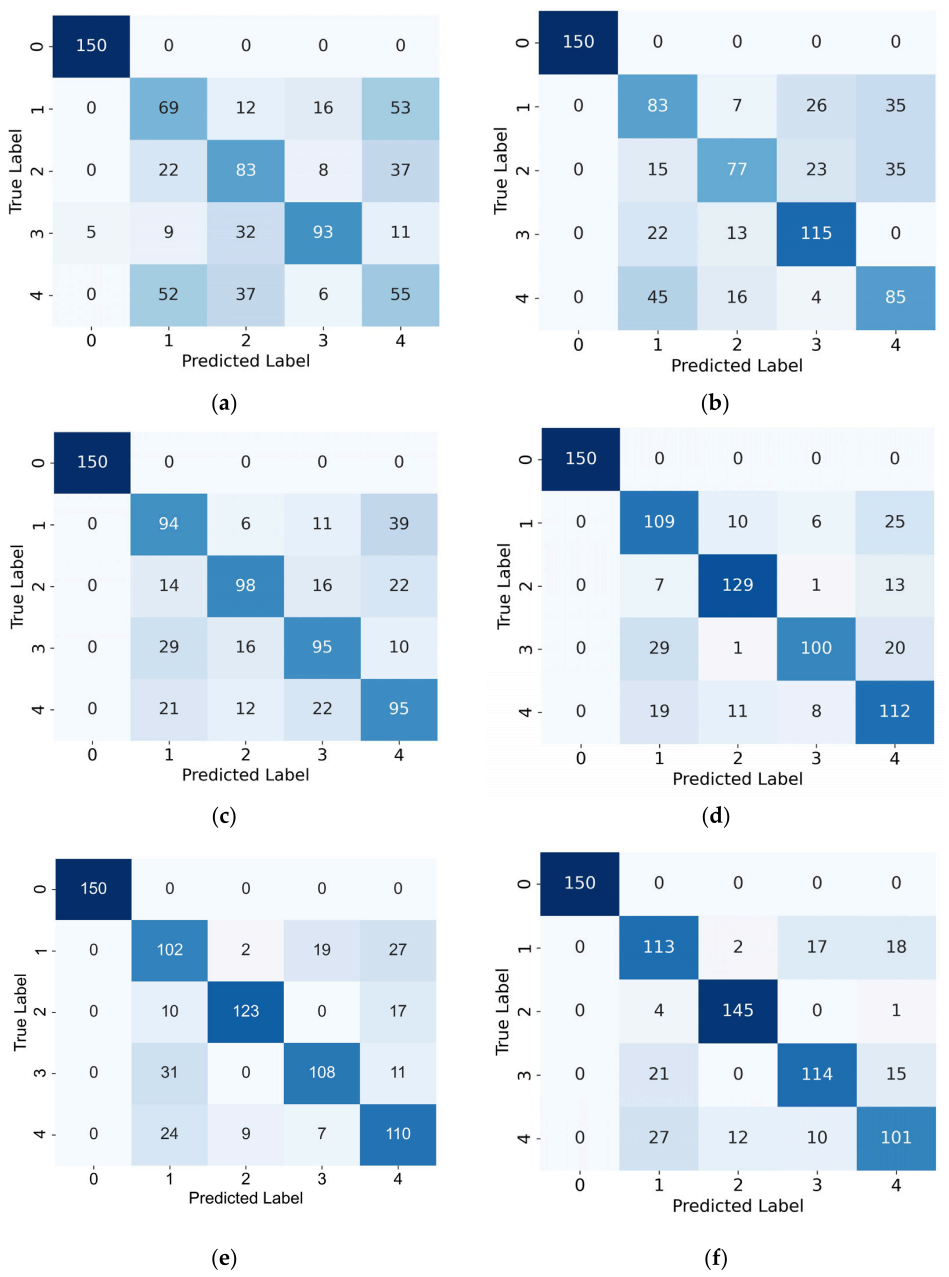
Confusion matrices for the user 1 model across the various methods in diagnostic task T2-un: (**a**) Local method; (**b**) Central method; (**c**) FedAvg method; (**d**) LFDG method; (**e**) FDDG method; (**f**) FedGen method.

To further analyze the classification differences across categories and the generalization advantages of the proposed method, Figure 7 shows the test confusion matrices for user 1’s model across the various methods in diagnostic task T2-un. The values on the main diagonal of the confusion matrix represent the number of samples correctly classified into each category. To quantitatively evaluate the performance across different fault types, Table 3 further reports the per-class recall, balanced accuracy, and Macro-F1 derived from these confusion matrices. Notably, the significant differences in performance among the various methods are primarily concentrated in the identification of specific faulty samples. Specifically, the Local model showed severe misclassification in category 1 and category 4, with a recognition accuracy of less than 40% for category 4 in particular. As quantified in Table 3, FedGen achieves the highest Macro-F1 (91.2%) and balanced accuracy (91.2%), outperforming LFDGN (88.9% and 89.3%, respectively). Furthermore, taking the identification of fault category 2 as an example, the best performance achieved by the comparison method was only 86% (achieved by LFDG), whereas the FedGen method significantly improved this to 96.7%. On the challenging category 4, FedGen attains 67.3% recall, which is significantly higher than Local (36.7%) and FedAvg (62.0%), though still leaving room for improvement. These results provide further intuitive validation of the proposed method’s reliable feature learning and knowledge generalization capabilities under complex distribution shifts.

### 5.2. Feature Visualization Analysis

**Figure 8:**
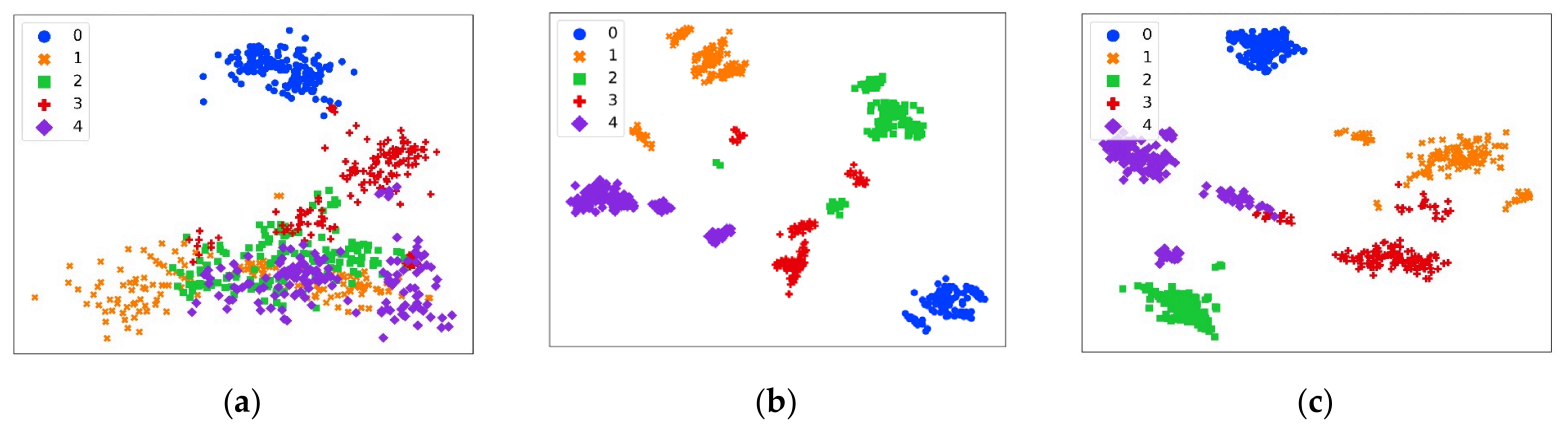
Feature distribution comparison of test results of user 1 model with local and FedGen methods on diagnosis task T2-un: (**a**) Local method; (**b**) FedAvg method; (**c**) FedGen method.

To further illustrate the performance of the proposed method at the feature learning level and to explain the role of virtual SI in the model’s discriminative ability, we employ t-SNE to visualize the high-dimensional features extracted by the model in two dimensions. Figure 8 compares the distribution of features extracted by the Local method and FedGen method during the user 1 model testing process in diagnostic task T2-un. All methods adopt identical t-SNE configurations: perplexity = 30, learning rate = 1000, and 1000 iterations, with the same random initialization shared across all methods. To quantitatively evaluate the separability of features, we calculated the ratio of intra-class distance to inter-class distance in the original feature space: Local scored 1.00 (the normalized baseline), FedAvg scored 0.58, and FedGen scored 0.25.

Observation shows that, since the Local method relies only on training with local data, it struggles to overcome domain drift caused by unknown working conditions. This results in severe feature space overlap among different fault categories (particularly between category 1 and category 4), leading to highly ambiguous decision boundaries. In contrast, the feature distributions generated by the FedGen method show better intra-class compactness and inter-class separation. This is because FedGen uses virtual SI to generate augmented features that simulate changes in the latent distribution, which are then used to enhance model training, thereby guiding the model to learn domain-invariant features. This strategy essentially expands the robustness boundary within the feature space, significantly increasing the margin at the decision boundary for different fault categories, thereby robustly improving the model’s classification accuracy under unknown working conditions.

### 5.3. Ablation Study

To evaluate the individual contributions of the proposed modules, a comprehensive ablation study is conducted by designing four distinct variants (M1 to M4). Specifically, M1 acts as a baseline that removes the statistical manifold generation and covariance expansion modules, relying solely on the exchange of raw real SI among existing users. M2 retains GMM fitting but disables covariance expansion by fixing *λ* = 1.0, M3 preserves GMM and expansion while replacing the difference-aware assignment with random allocation, and M4 removes the JS consistency regularization term.

**Figure 9:**
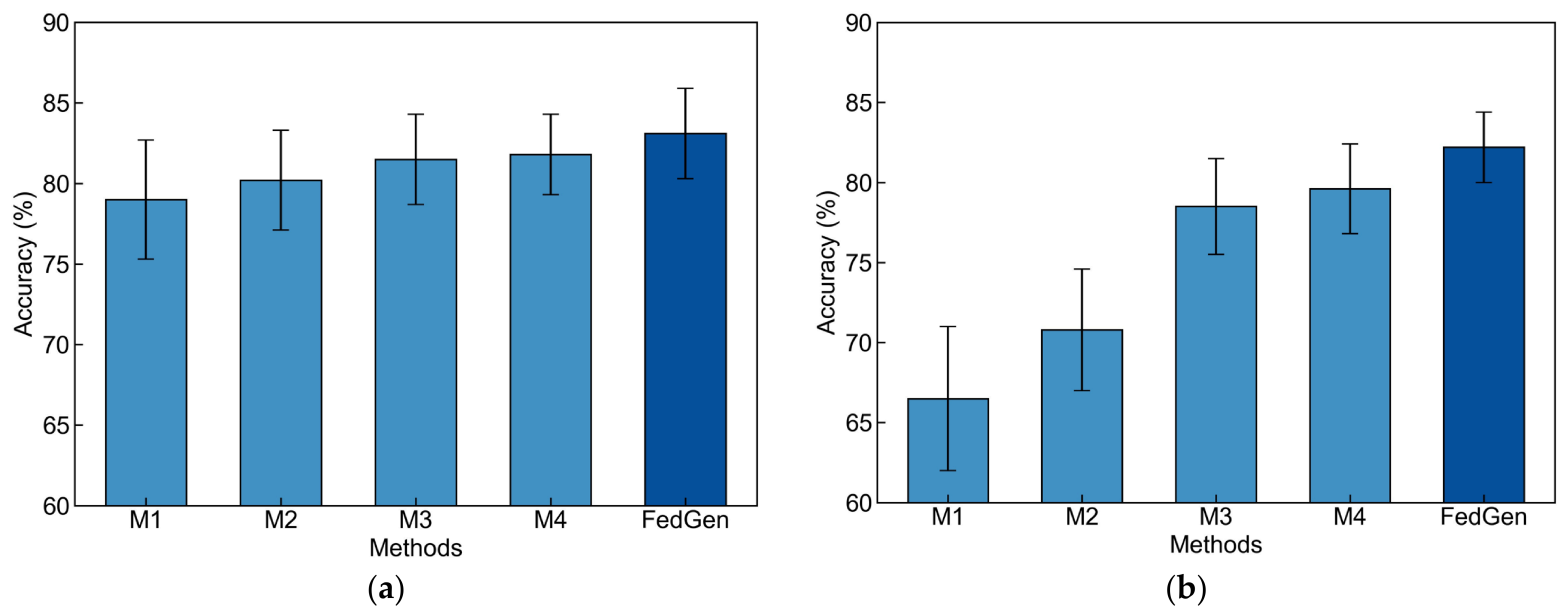
Ablation study on Task T2-un and T5-un: (**a**) Task T2-un; (**b**) Task T5-un.

As shown in Figure 9, the ablation results on Tasks T2-un and T5-un quantitatively validate the effectiveness of each component within the proposed framework. The most significant performance degradation is observed in the M1 variant. This confirms that directly exchanging raw statistics is insufficient to cover unknown working conditions. The M2 variant shows limited improvements, indicating that covariance expansion is the critical enabler for extrapolating beyond the convex hull of source distribution. M3 yields degraded performance with increased standard deviations compared to the FedGen, demonstrating that the difference-aware assignment mechanism contributes not only to accuracy improvement but also to stability. M4 exhibits a relatively moderate drop, suggesting that JS consistency regularization plays an auxiliary role in stabilizing the decision boundary. Finally, the complete FedGen achieves the highest accuracies with the lowest standard deviations, demonstrating that the synergistic combination of manifold modeling, covariance expansion, difference-aware assignment, and consistency regularization is essential for robust and stable cross-condition fault diagnosis.

### 5.4. Parameter Sensitivity Analysis

**Figure 10:**
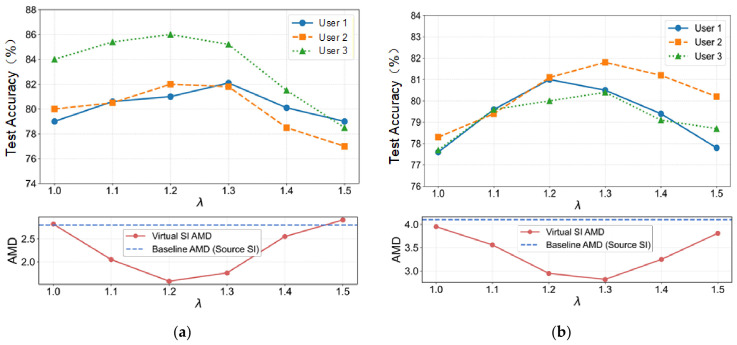
Sensitivity analysis *λ* on Task T2-un and T5-un: (**a**) Task T2-un; (**b**) Task T5-un.

To assess the specific influence of the expansion coefficient *λ* on the model’s generalization performance, parameter sensitivity analyses were conducted under tasks T2-un and T5-un, with the results shown in Figure 10. The results indicate that as *λ* gradually increases from 1.0 to 1.5, the diagnostic accuracy of each user model generally follows a trend of first increasing and then decreasing.

Specifically, the performance of the user model generally reaches its optimal level within the range *λ* ∈ [1.1, 1.3]. This phenomenon suggests that moderate covariance expansion can reasonably broaden the uncertainty boundaries of the distribution in the statistical manifold space, enabling the generated virtual features to effectively simulate the data distribution under unknown working conditions, thereby enhancing the model’s generalization ability. When the value of *λ* is small (such as *λ* = 1.0), boundary extrapolation is too conservative and difficult to substantially bridge the distribution shift between the source domain and the target domain; when *λ* is too large (such as *λ* > 1.4), excessive inflation will introduce a large amount of statistical noise, generating samples that do not conform to physical laws and leading to a significant decline in diagnostic performance.

To further verify the ability of the generated SI to cover potential unknown working conditions, we introduced the average minimum distance (*AMD*) metric. *AMD* effectively measures the coverage of the generated boundaries over the target domain by calculating the distance from each real target sample to its nearest neighbor in the generated SI. It is defined as:

(11) $$AMD\left(\lambda \right)=\frac{1}{\left|U_{T}\right|}\sum_{u_{v}\in U_{T}}\underset{u_{v}\in U_{\lambda }}{\min}\|u_{t}-u_{v}{\|}_{2}$$

where *U_T_* is the target-domain SI set, and *U_λ_* is the virtual SI set generated with expansion coefficient *λ*. A fixed baseline is computed as the average minimum distance from the target SI to the original source SI set *U_S_*:

(12) $$AMD_{src}=\frac{1}{\left|U_{T}\right|}\sum_{u_{v}\in U_{T}}\underset{u_{s}\in U_{S}}{\min}\|u_{t}-u_{s}{\|}_{2}$$

This baseline remains constant across *λ* and is depicted as a horizontal dashed line.

As shown in Figure 10, as *λ* increases to 1.1–1.3, *AMD* drops sharply, which confirms that an appropriate covariance expansion can successfully cover unknown working conditions. It is worth noting that the above trend is consistent across the bearing (T2) and gearbox (T5) tasks, validating the parameter’s robustness across different machine types and generalization scenarios.

**Figure 11:**
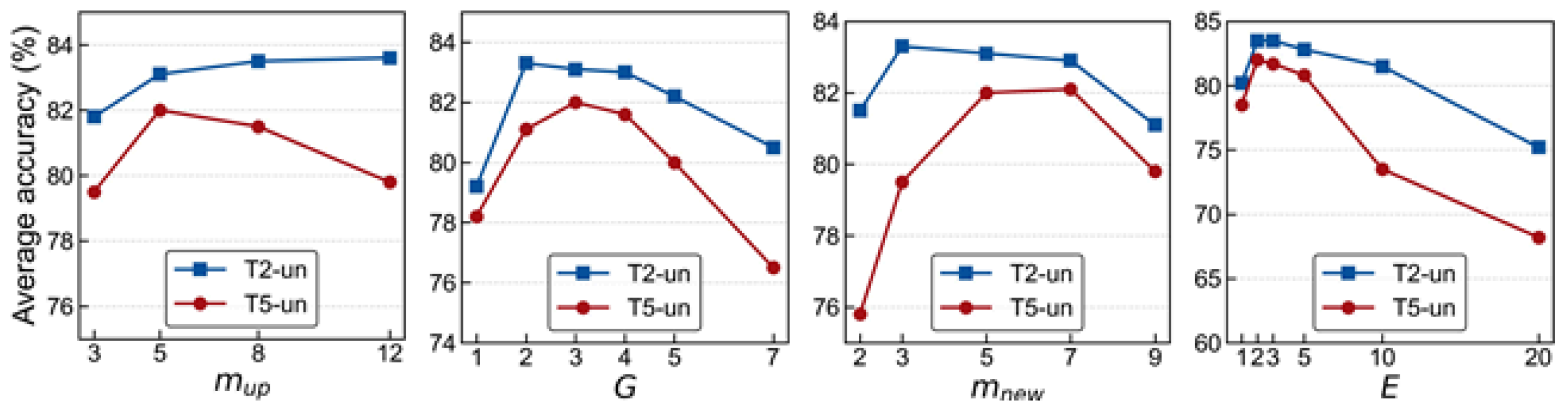
Sensitivity analysis (*m*_up_, *G*, *m*_new_, *E*) on Task T2-un and T5-un.

Furthermore, we conducted sensitivity analyses on key hyperparameters, including the uploaded SI count *m*_up_, the number of GMM components *G*, the assigned SI count *m*_new_, and the communication interval *E* (the number of communication rounds is fixed at 100). As shown in Figure 11, regarding *m*_up_, when the number increased from 3 to 5, the accuracy of both tasks improved significantly, indicating that an adequate number of SI samples is crucial for GMM fitting; as the number continued to increase, T2 approached saturation, while T5 began to decline due to the introduction of noise. For *G*, the results indicate that the optimal range is 2–5, as increasing *G* tends to lead to overfitting. Regarding *m*_new_, T2-un achieves optimal performance when *m*_new_ = 3, while T5-un achieves optimal performance when *m*_new_ = 5–7. This is because a *m*_new_ that is too small results in the generated virtual features failing to cover a sufficient range of working conditions, thereby limiting the generalization gain; conversely, a *m*_new_ that is too large reduces the number of virtual features per type, which can easily confuse the model. Regarding *E*, when *E* is 1, accuracy decreases because the exchange occurs before the SI has stabilized; when *E* ≥ 5, performance deteriorates sharply due to delayed coordination.

### 5.5. Computational and Communication Efficiency

To verify the feasibility of deploying the proposed methods in resource-constrained Industrial Internet of Things (IIoT) environments, this section provides a quantitative evaluation of the communication overhead and computational efficiency of different methods. The results are shown in Table 4.

In terms of communication overhead, FedAvg requires the transmission of the full model parameters at each iteration, with a single communication payload of around 37.4 MB. In contrast, the proposed FedGen only needs to exchange low-dimensional feature SI, effectively reducing the single communication payload to 1.25 KB. This low bandwidth requirement makes it more suitable for deployment in narrowband industrial networks such as LoRa and NB-IoT. In terms of computational efficiency, the total local training time of FedGen (about 14 min) is slightly higher than FedAvg (about 8 min). This is mainly due to the additional computational overhead introduced by the extraction of local feature SI and the reconstruction of virtual features, but this time cost is within a reasonable acceptable range during the offline training phase. Furthermore, during the testing phase, the single-sample inference latency for all methods was less than 2 ms, indicating that the generative feature enhancement strategy did not increase the computational load of the model during online deployment and is fully capable of meeting the real-time monitoring requirements in industrial scenarios.

Furthermore, we theoretically analyze the complexity of FedGen’s core operations. Locally, representative SI mining incurs a complexity of $O(m_{\mathrm{up}}\cdot |Q|\cdot D)$, where *m*_up_, |*Q*|, and *D* denote the uploaded SI count, local pool size, and feature dimension, respectively. On the cloud server, the GMM fitting complexity per EM iteration is bounded by $O(N_{\mathrm{up}}\cdot G\cdot D)$. Because the total uploaded SI (*N*_up_) is strictly proportional to *K*, this server-side cost scales linearly with the user scale. Finally, the upper bound on the complexity of the personalized SI assignment is $O(m_{new}\cdot |U|\cdot D^{2})$. The above analysis shows that as the user scale expands, the additional computational burden on FedGen remains within manageable limits.

### 5.6. Limitations and Future Work

While FedGen achieves robust generalization, several avenues for further refinement remain. Firstly, regarding physical interpretability, although SI is considered an effective carrier of domain features based on instance normalization theory, a clear quantitative mapping between SI and specific physical parameters such as speed or load has not yet been fully established. Second, concerning data leakage risks, although SI reduces privacy exposure, the theoretical possibility of reconstructing raw data distributions from statistical summaries persists. Third, in terms of data distribution, the current method assumes relatively balanced class distributions and identical label spaces across all clients. However, in real IIoT deployments, scenarios such as severe class imbalance, partially overlapping fault categories across clients (e.g., some clients covering only a subset of fault types), significant data quality disparity (e.g., noisier measurements from certain users), and extreme distributional deviation of a single user are common. These factors may compromise the SI modeling accuracy for minority fault categories or introduce bias in GMM manifold fitting. Finally, λ selection currently relies on empirical sweeping. Addressing these challenges in the context of privacy-preserving diagnostics is the focus of our future work.

## 6. Conclusions

To address the challenges of data privacy and generalization under unknown working conditions in IIoT scenarios, this paper proposes a collaborative fault diagnosis method (FedGen). This method establishes a closed-loop collaborative framework driven by feature statistical information. By integrating Gaussian mixture modeling with a covariance expansion strategy, FedGen successfully reconstructs and extrapolates the latent statistical manifold of multi-source data. This generative mechanism actively simulates the continuous evolution of working conditions, providing diverse virtual features to cover unknown target domains. Coupled with difference-aware assignment and local instance normalization, the strategy effectively decouples fault-essential features from working condition variations, significantly enhancing the model’s out-of-distribution robustness.

The comprehensive experimental results indicate that, when dealing with completely unknown test conditions, FedGen outperforms existing mainstream methods in terms of diagnostic performance and robustness. At the same time, the efficiency analysis demonstrates FedGen’s extremely low communication requirements and controllable local computational overhead, proving that it is highly suited to the engineering requirements of edge computing scenarios, which demand real-time monitoring and lightweight deployment. Given its data-driven nature and reliance on generic sensor signal statistics, the proposed method is conceptually applicable to other industrial assets equipped with vibration or condition monitoring systems, such as motors, pumps, compressors, and turbines.

## Figures and Tables

**Table 1:** Diagnosis tasks of dataset A.

Task	T1-cro, T1-un		T2-cro, T2-un		T3-cro, T3-un		T4-cro, T4-un	
	Training	Test	Training	Test	Training	Test	Training	Test
User 1	A1	(A2, A3), A4	A1	(A2, A4), A3	A1	(A3, A4), A2	A2	(A3, A4), A1
User 2	A2	(A1, A3), A4	A2	(A1, A4), A3	A3	(A1, A4), A2	A3	(A2, A4), A1
User 3	A3	(A1, A2), A4	A4	(A1, A2), A3	A4	(A1, A3), A2	A4	(A2, A3), A1

**Table 2:** Diagnosis tasks of dataset B.

Task	T5-cro, T5-un		T6-cro, T6-un		T7-cro, T7-un		T8-cro, T8-un	
	Training	Test	Training	Test	Training	Test	Training	Test
User 1	B1	(B2, B3), B4	B1	(B2, B4), B3	B1	(B3, B4), B2	B2	(B3, B4), B1
User 2	B2	(B1, B3), B4	B2	(B1, B4), B3	B3	(B1, B4), B2	B3	(B2, B4), B1
User 3	B3	(B1, B2), B4	B4	(B1, B2), B3	B4	(B1, B3), B2	B4	(B2, B3), B1

**Table 3:** Macro-F1, balanced accuracy, and per-class recall of all methods on task T2-un.

	Local	Central	FedAvg	LFDGN	FDDG	FedGen
C0-recall	100.0	100.0	100.0	100.0	100.0	100.0
C1-recall	46.0	55.3	62.7	72.7	68.0	75.3
C2-recall	55.3	51.3	65.3	86.0	82.0	96.7
C3-recall	62.0	76.7	63.3	66.7	72.0	76.0
C4-recall	36.7	56.7	63.3	74.7	73.3	67.3
Bal. Acc.	60.0	68.0	70.9	80.0	79.1	83.1
Macro-F1	60.2	67.9	71.0	80.1	79.3	83.0

**Table 4:** Computational and communication costs of different methods.

Method	Communication Content	Single Communication Packet Size	Total Local Training Time	Inference Time
FedAvg	Complete model parameters	~37.4 MB	~8 min	<2 ms
LFDGN	Category prototype	~10.00 KB	~10 min	<2 ms
FedGen	Feature SI	~1.25 KB	~14 min	<2 ms

## Data Availability

Dataset available on request from the authors. The raw data supporting the conclusions of this article will be made available by the authors on request.

## Abbreviations

The following abbreviations are used in this manuscript:

FDG	Federated Domain Generalization
FedAvg	Federated Averaging
FedGen	Federated Generalization Fault Diagnosis
FedSI	Federated Statistical Information
FL	Federated Learning
GMM	Gaussian Mixture Model
IN	Instance Normalization
OOD	Out-of-distribution
RBF	Radial Basis Function
SI	Statistical Information
SIe	SI exemplars
SMGPA	Statistical Manifold Generation and SI Personalized Assignment
t-SNE	t-distributed Stochastic Neighbor Embedding
